# A critical examination of empowerment discourse in medical tourism: the case of the dental tourism industry in Los Algodones, Mexico

**DOI:** 10.1186/s12992-018-0392-3

**Published:** 2018-07-20

**Authors:** Krystyna Adams, Jeremy Snyder, Valorie A. Crooks, Nicole S. Berry

**Affiliations:** 1 Faculty of Health Sciences, Simon Fraser University, 8888 University Drive, Burnaby, BC V5A 1S6 Canada; 2 Department of Geography, Simon Fraser University, 8888 University Drive, Burnaby, BC V5A 1S6 Canada

**Keywords:** Medical tourism, Dental tourism, Critical discourse analysis, Health equity

## Abstract

**Background:**

Medical tourism is a term used to describe the phenomenon of individuals intentionally traveling across national borders to privately purchase medical care. The medical tourism industry has been portrayed in the media as an “escape valve” providing alternative care options as a result of vast economic asymmetries between the global north and global south and the flexible regulatory environment in which care is provided to medical tourists. Discourse suggesting the medical tourism industry necessarily enhances access to medical care has been employed by industry stakeholders to promote continued expansion of the industry; however, it remains unknown how this discourse informs industry practices on the ground. Using case study methodology, this research examines the perspectives and experiences of industry stakeholders working and living in a dental tourism industry site in northern Mexico to develop a better understanding of the ways in which common discourses of the industry are taken up or resisted by various industry stakeholders and the possible implications of these practices on health equity.

**Results:**

Interview discussions with a range of industry stakeholders suggest that care provision in this particular location enables international patients to access high quality dental care at more affordable prices than typically available in their home countries. However, interview participants also raised concerns about the quality of care provided to medical tourists and poor access to needed care amongst local populations. These concerns disrupt discourses about the positive health impacts of the industry commonly circulated by industry stakeholders positioned to profit from these unjust industry practices.

**Conclusions:**

We argue in this paper that elite industry stakeholders in our case site took up discourses of medical tourism as enhancing access to care in ways that mask health equity concerns for the industry and justify particular industry activities despite health equity concerns for these practices. This research provides new insight into the ways in which the medical tourism industry raises ethical concern and the structures of power informing unethical practices.

**Electronic supplementary material:**

The online version of this article (10.1186/s12992-018-0392-3) contains supplementary material, which is available to authorized users.

## Background

Medical tourism is a term used to describe the phenomenon of individuals intentionally traveling across national borders to privately purchase medical care. Individuals might be motivated to travel as medical tourists to avoid wait lists for care and/or to access more affordable care than is domestically available or care not typically available within their domestic health care system [1]. Research on this phenomenon suggests that medical tourists travel between and within both the global north and global south; however, the majority of media and academic attention has focused on the flow of patients from the global north accessing care in the global south care that is more affordable than in their home countries [2, 3]. Dental tourism is one type of medical tourism characterized by individuals purchasing dental care outside of their home country and paying for the dental procedures out-of-pocket [4].

Media and industry portrayals of medical tourism, including dental tourism, often suggest that this practice enables both patients and providers to choose amongst a variety of industry sites and regulatory environments to arrange their care or work experiences [5, 6]. The industry has been portrayed in the media as a “safety zone” or “escape valve” providing alternative care options as a result of vast economic asymmetries between the global north and global south and the flexible regulatory environment in which care is provided to medical tourists ([7], p. 1848;[8]). For example, media reports on the dental tourism industry in Los Algodones indicate that patients can shop around for care within a “dental oasis” of clustered care providers, enabling patients to easily try out different providers and negotiate the care and price that fits their needs [9]. Research has also demonstrated how elite industry stakeholders including owners of large medical clinics, patient facilitation companies, and governmental bodies have encouraged the relaxation of regulatory mechanisms to enable lower cost care provision, entice medical providers keen to offer certain types of care that might not be allowed in other jurisdictions, and ultimately increase the flow of medical tourists and profits to that particular site [10, 11]. In this paper, we refer to the common discourse presented in the media of the medical tourism industry as both enhancing access to medical care and driving economic development for industry sites as a discourse of empowerment.

There are good reasons to be critical of this discourse of empowerment, however. Drawing on critiques of privatization of health care encouraged by neoliberal discourses and policies [12, 13], the medical tourism literature has demonstrated how industry stakeholders have taken up neoliberal assumptions about the role of market-driven care in driving economic development and enhancing patient access to care to push forward an overly simplistic discourse of empowerment in medical tourism [14, 15]. For example, the alternative care options promoted in the media and industry sources of information are mostly taken up by patients from the global north and only those who are able to travel and pay for care, limiting this empowerment to a particular population which does not include the most vulnerable patients struggling to access care [10]. Furthermore, by presenting choice in health care as necessarily empowering [8], media coverage on medical tourism and industry discourse ignores and dismisses the myriad ways in which more choice for patients and providers can be “stressful, confusing, time consuming, and impossible to do well” ([16], p. 41). Researchers suggest choice rhetoric might be taken up in media discourse and health system planning in ways that assume more choice is always good while dismissing or ignoring the unintended consequences of policies focused on enhancing patient and provider choice. These unintended consequences include new safety risks for patients when more care choices hinder effective regulatory mechanisms and/or the irresponsible use of health care resources when care provision intent on enhancing choice does not align with the health care needs in the community [16, 17].

Discourse emphasizing medical tourism activities as necessarily empowering care providers and other industry employees also fails to consider how the economic development opportunities promised by proponents of industry development are differentially experienced by stakeholders occupying a range of positions in the industry. Many entrepreneurial providers have struggled to guarantee profits when competing for the same pool of patients in the global medical tourism industry [18, 19] while the relaxation of regulatory mechanisms such as licensing requirements for medical professionals and professional regulation could also enable and exacerbate unjust practices such as low wages for industry employees and limited professional oversight to promote responsible usage of health resources [8, 20]. Overall, these critiques emphasize the potential health equity and health system implications of medical tourism, ethical concerns which are often dismissed in media coverage in favour of a discourse of empowerment.

In the remainder of this article, we examine how the circulation of empowerment discourse amongst medical tourism industry stakeholders can work to mask or dismiss ethical concerns for the industry, enabling ongoing industry development with limited regulation or measures to mitigate negative health equity implications of and safety concerns for the industry. Drawing on a case study of dental tourism practices within the industry site of Los Algodones, Mexico, we provide a critical examination of how and why a discourse of empowerment is taken up by industry stakeholders and the ways in which this discourse serves to further entrench structural injustices such as poor access to health care and unfair labour conditions. We also draw on this case study to question whether providing more choice to patients is necessarily desirable for building robust health care systems if this choice involves limiting regulations needed to protect patient safety and responsible use of finite health resources. Before we examine this localized example in more detail, we first provide an overview of medical tourism industry development in Mexico and discuss why we chose the dental tourism industry in Los Algodones as our site of inquiry.

## Dental tourism in Los Algodones, Mexico

Los Algodones is a small town located directly across the border from Yuma, Arizona in the United States. This location has informed the development of a robust dental tourism industry and encouraged slow but increasing growth of the town’s population. Currently, approximately 500 dentists work in Los Algodones, which is a town of 6000 residents. As a result of this concentration of dental care providers, Los Algodones has earned the title of “dental capital of the world” according to media reports [21, 22]. This exceptional concentration of dentists has been characterised in the media and in industry marketing and promotional materials as empowering patients though increased choice for affordable and acceptable dental care than is typically available in visitors’ home care contexts [23]. Dentists in Los Algodones primarily treat American and Canadian patients, many of whom are seniors who spend the winter months in Yuma as ‘snowbirds’. Dental tourism in this site relies on the concentration of dental clinics available to treat tourists crossing the border for the day as well as affiliated staff and businesses including numerous labs, street promoters, patient facilitators, and medical repositories to attract customers and facilitate care provision to a high volume of patients. Generally, patients travel to Los Algodones with the intention of purchasing more affordable care than is available in their home countries; however, some tourists decide to become patients while already visiting Los Algodones to souvenir shop, enjoy the bars and restaurants, purchase lower cost alcohol or prescription drugs than available back home, or participate in community events [22, 24].

Los Algodones is just one of many sites in Mexico catering to medical tourists from the global north. National policies and contexts seemingly play an important role in shaping industry practices and policies in sites such as Los Algodones [11, 21]. In Mexico, the federal, state, and municipal governments have contributed substantial resources towards promoting and financially supporting tourism activities, including medical tourism [25]. The Mexican Tourism and Convention Bureau (COTUCO) has listed medical tourism as one of 12 categories of tourism diversifications being developed and promoted in Mexico (VisitMexico.com). The state of Baja California, where Los Algodones is situated, has a webpage dedicated to advising prospective medical tourists on what to expect when traveling to the state as a medical tourist and how to choose destination cities, providers, and procedures during their medical tourism experience (Bajahealthtourism.com). Overall, the medical tourism industry in northern Mexico is portrayed on many of these government websites and promotional materials as both an important economic activity encouraging entrepreneurial pursuits as well as an opportunity to enhance patient access to desired care. As the Baja California webpage devoted to medical tourism states: “For millions, medical tourism has become the healthy choice”, exemplifying how medical tourism in this context is framed as necessarily enhancing health outcomes for a large proportion of the global population (Bajahealthtourism.com). We contend that this framing ignores and renders invisible those who cannot participate in this “healthy choice”, a concern which drives our analysis below.

## Methods

We employed case study methodology to examine the dental tourism industry in Los Algodones through interviews, media reviews, and first-hand observation. Case study methodology enables a nuanced examination of a well-defined and bounded phenomenon to provide insight into complex social relations and processes [26, 27]. While dental tourism occurs in other northern Mexican border towns, Los Algodones is unique in that it is the only medical tourism destination site in this region focused almost entirely on dental care instead of a range of medical care options. Our case study of this unique industry site aims to trace how global forces shape local industry practices to develop a better understanding of ethical concerns for medical tourism practices grounded in the lived experiences of individuals working and residing within a particular site.

To gain insight into individuals’ experiences of industry practices in Los Algodones, we recorded 43 semi-structured interviews with individuals representing a range of different professional roles including dentist (*n* = 30; nine women, 21 men), dental assistant (*n* = 4; two women, two men); street promoter or patient facilitator (n = 4; all men); marketing professional and business executive (n = 3; one woman, two men); and municipal tourism planner (*n* = 2; all men). Interviews were conducted during 3 months of observational field work in Los Algodones. KA conducted all interviews in English, except for six which I conducted in Spanish. During the interviews, participants were asked about their decisions to work in the industry, descriptions of their professional role and influences on their work and industry practices, and any concerns for current industry practices and industry development. Specific questions were organized by a semi-structured guide (see Additional file 1) that allowed participants to raise issues and concerns not initially probed. The recorded interviews were then transcribed verbatim and interviews conducted in Spanish were transcribed and translated to English by a native Spanish speaker for ease of analysis. Recorded interviews lasted between 20 and 90 min with the majority lasting approximately 45 min.

To analyze the data, I conducted a thematic review along with my supervisory committee of interviews and informal conversations with industry employees working in Los Algodones. All investigators independently reviewed six transcripts and we met as a team to reach consensus on significant emergent themes and the scope and scale of each. One of the themes identified through this process was provider perceptions of empowerment, both for themselves and for patients. The first author then developed a coding scheme to capture and organize the key issues informing this theme and then implemented coding with the assistance of the NVivo data management program. Data extracts related to “empowerment discourse” were compiled and reviewed to identify key sub-themes and contrast them against relevant published literatures. Throughout the analytic process we enhanced rigour through employing investigator triangulation at multiple points, identifying necessary context relevant to our interpretation in order to enhance transferability, and facilitating trustworthiness by triangulating observational field notes with interview data [28].

## Results

In the remainder of this section, we share the findings from our interviews and highlight care provider depictions of practices characterized as empowering for providers and patients while also drawing attention to participant concerns for various industry practices. These findings highlight the ways in which a discourse of empowerment justifies the largely private provision of dental care in Los Algodones, and particularly to foreign tourists, despite safety and health equity concerns for the industry as described by several participants.

### Provider (dis)empowerment

The majority of participants spoke very positively about the dental tourism industry in Los Algodones and hoped to continue working in the industry. Participants suggested that they chose to work in this industry because it afforded better working conditions than other alternatives, including wages, unique professional development, and opportunities to provide the types of care for which they were trained. Participants generally agreed that it would be very difficult to find work as a dentist in both the public and private sectors in certain regions of Mexico due to limited demand and job positions, potentially pushing dentists to seek work in the dental tourism industry. Some participants suggested that intense competition for employment in the dental sector might be caused by accepting too many dentists into dental colleges. Other participants insisted that competition for work amongst dentists is a result of structural barriers to accessing dental care in Mexico, with one participant stating: “It has to do with poverty, if you do not have money to get dressed or to eat you do not have money to go to the doctor, or to buy medicines”. This participant suggested that dentists from poorer regions of Mexico might seek employment in other regions, including dental tourism sites, where tourists from the global north can more easily pay for private medical care.

Participants suggested that dentists might be drawn to work in the industry not only for better wages but also because they preferred the working conditions and professional opportunities afforded by the industry. For example, one participant indicated that she decided to work in the dental tourism industry due to her struggle to balance work with child care. She explained that dental tourists are typically retired and are willing to book appointments during the day, unlike local residents who typically have work commitments requiring them to book appointments during evenings and weekends. Participants highlighted other pull factors for working in the industry including financial investment by government and businesses supporting medical tourism industry development. Participants suggested that this investment enabled dentists to open their own clinics with better equipment and patient support staff, as exemplified by the following quote:

Okay the situation in the late 90’s, we were around 30, 40 dentists. And then the boom from the place start noise in everywhere and more people they start coming down, until the investment people or the investment in dental marketing start establish here. People who have money start opening clinics and then contract doctors. (dentist).

Because dental tourists typically access a high volume of care in a shorter amount of time compared to local residents, this has allowed dentists to treat more patients and develop their skills. Many participants explained that they prefer treating foreign patients, and almost exclusively treat non-Mexican patients, because they can provide the treatment they were trained to do, enhancing their professional development. One participant stated that: “There are two cultural levels. This is local culture, this is Mexican culture. We work by appointments, we have medical records, we have to check everything, x-ray machines and everything. They [Mexican patients] want to pull it, pull the tooth out [without proper examinations first]. Not everybody, but 90%” (dental clinic owner), suggesting that the type of care provided in the clinics catering to dental tourists is distinct from care provision in Mexico outside of the industry. Many participants also distinguished their experiences working within the medical tourism industry and outside of this industry by referring to the amount of money provided by dental clinic owners in Los Algodones to attend conferences, ongoing education, and other forms of professional development.

Despite generally positive portrayals from dentists about their work in the industry, many participants also shared stories that contradicted common assertions that the industry is necessarily characterized by professional development and desirable employment opportunities. For example, many participants explained that most dentists working in Los Algodones were not part of the local professional dental association despite professional guidelines requiring dentists to join a professional association if practicing in Mexico. While most participants did not express concerns about the limited participation in Los Algodones’ professional association, referring to consumer-driven forms of regulation as encouraging acceptable quality of care, some participants raised concerns about how limited professional regulation enables unethical or unsafe dental practice. This concern was particularly focused on “some of the new entrepreneurs [who] don’t want to have to pay [for professional association membership] because they want to sell their dental care for even cheaper” (dentist). Participants who raised concerns about this limited participation in the local professional association typically emphasized competition between providers as limiting communication between different medical professionals in Los Algodones, frustrating some participants who felt better collaboration at the professional association or other venues is necessary to promote high standards of care provision.

Some participants suggested that even if dentists wanted to join the professional association or other professional development activities, sometimes this is not possible due the long hours they work treating a high volume of patients. One participant explained that “The thing is, we have too many people. For example, if they sit in the chair, sometimes I don’t stand up until 10:00 pm so I don’t know. I don’t know if this guy has 2 or 3 years since he did the training” (dentist). Other participants expressed similar concerns that with long working hours, many dentists do not have time to join the professional organization and also cannot provide peer-regulation as there is limited opportunity to contact and communicate with dentists working in different clinics.

A few participants questioned assumptions that all dentists can access professional development opportunities to improve their skills by highlighting how some of the clinic owners do not have a dental background and as a result, might be reluctant to fund or enable dentists to attend professional development activities. This concern is exemplified by the following quote:

Yes, there are people interested in this [regulation], but nobody pays attention to us. The industry should be regulated; it would help a lot to have control over situations that are currently happening here. For example, because everything here is driven by money, people who sold blankets in stores before and now have dental clinics, they do not want to sell blankets anymore, what they do is to put a dental chair and someone to work there and now they are the owners of a clinic that is not regulated. That is why there should be regulation. (dentist).

This quote demonstrates a common perspective from dentists we interviewed that employment in the industry varies considerably between clinics, especially in terms of access to professional development opportunities and standards of care. Participants who raised a similar concern emphasized that despite the existence of high quality care in Los Algodones, there are providers who provide lower standards of care, indicating that this is made possible by the limited enforcement of regulatory standards.

### Patient (dis)empowerment

Within a context of unique professional development opportunities and investment in high quality care provision, many participants portrayed their role as dentists working in Los Algodones in a very altruistic way and emphasized how their services expand access to desired dental care for populations facing structural barriers to accessing this care. This perception was largely informed by providers’ interactions with patients who expressed high levels of gratitude for the care they had received. Multiple participants emphasized the concentration of dental clinics and affiliated services in Los Algodones as producing a type of medical marketplace for dental care that enables patients to shop around for care and select services in a clinic that best fits their needs. As one participant explained that:

Well, it’s a small town, it has only two blocks actually, and you can find about 310 dental clinics, at least. And they call it “molar city”, people in the United States call it “molar city” ‘cause people come to get their teeth done [...] So everybody is happy here. (municipal tourism planner).

This quote exemplifies common participant perspectives that the concentration of dentists working in Los Algodones has produced a unique care context characterized by the high concentration of health human resources. Additionally, some clinic owners have hired patient coordinators and other support staff to respond to patient inquiries “around the clock”, enhancing patients’ ability to access care that meets their needs.

Participants suggested that the volume of online resources available to dental tourists enhances patients’ access to desired care. Participants indicated that many dental tourists use online review websites to compare patient perspectives of different clinics and dental tourism industry sites in ways that inform patients of a variety of choices available to them in Los Algodones. Many participants also suggested that consumer-driven forms of regulation and competition for patients necessarily enhance quality of care because patient reviews online ensure “bad doctors” (dental clinic owner) go out of business. According to participants, this review system encourages dentists to provide high quality care because they know that patients can provide a negative review online and will want to avoid harming their reputation. As a result, participants suggested that some clinic owners focus on meeting customer demands through changes to their practices. These changes can include hiring untrained technicians to lower their prices, adjusting clinic practices to speed up care (i.e., one clinic has patients move from room to room in an assembly-line fashion to reduce the time dentists spend moving between offices), and hiring patient coordinators or working with facilitation companies to arrange patients’ travel details and answer questions throughout their medical tourism experience.

Despite the common assertion from participants that the dental tourism industry in Los Algodones enhances patients’ choices for more accessible and affordable dental care than typically available in their home countries, particular anecdotes and perspectives disrupted this discourse by highlighting exclusionary practices in the industry and concerns for unethical and/or substandard care practices including unsafe care protocols and irresponsible use of health resources. Multiple participants questioned the effectiveness of patient review websites in driving acceptable standards of care in Los Algodones’ dental tourism industry. Participants described how some patients have published inaccurate or unfair reviews when they did not like their experience. The possibility of patients publishing negative reviews online has encouraged some care providers to give treatment according to patients’ demands or expectations, even if these demands do not align with safe and ethical treatment protocols. Many participants explained that dentists and other industry employees assume other employees will attract more customers by adapting or responding to patient demands, encouraging care providers to adjust to these demands, as exemplified by the following quote: “But in season they’re coming from Canada or USA, so they have not much time […] but they want all the work fast, fast, fast. And we need to do it. Somebody will do it.” (dentist).

Within this competitive industry context, participants suggested that clinic employees commonly devote time and resources to providing customer service and improving online patient reviews in ways that might limit time to actually provide care to patients in need. Participants suggested that clinic employees, including dentists, often spend time reviewing x-rays and providing estimates so patients can shop around for the best price before booking their dental tourism trip. Furthermore, many dental clinics in Los Algodones advertise free x-rays and we witnessed one dental tourist undergo three x-rays in 1 day to compare treatment prices before determining which clinic to use. Some participants also suggested that dental tourists will sometimes get treatment fixed from a different provider at the first sign of pain, even if this care is not needed. Overall, participants suggested that patient reviews might cause dental tourists to feel overwhelmed when navigating so many different opinions, resulting in patients accessing multiple free examinations and diagnoses or other forms of unnecessary care, wasting dentists’ and other clinic employees’ time and resources.

Some participants raised concerns about exclusionary care practices in Los Algodones as many clinics focus on treating customers who will maximize profits. A few participants emphasized a large need for dental care amongst vulnerable populations living in and around Los Algodones and expressed their disappointment that many clinic owners and dentists do not use their resources to improve the oral health of these populations. Some dentists indicated that they provide treatment at lower cost or for free for friends, family members, or other local residents; however, participants often emphasized that local populations might only be able to access care when clinic providers are not busy treating foreign patients while some souvenir vendors said that they are never able to get the care they need in Los Algodones. Some participants explained that they don’t treat some local populations because they perceived them to be “bad patients” (dentist), characterised as patients who are late for appointments, who don’t pay in full for treatments, or request to have their teeth pulled instead of paying for recommended care. These participants suggested that they prefer treating foreign patients who pay in full, in American dollars, and in a shortened timeframe, allowing them to generate more profit by treating a higher volume of patients who pay more for each treatment.

Finally, many participants justified their focus on treating foreign patients despite needs for dental care in the local population by explaining how local residents have existing access to dental care in the public or private sector. However, the quality of dental care provided in the public sector was commonly described by participants as “very poor” and, as a result, some of our participants felt that elite industry members could do more to enhance the oral health of local residents who cannot afford to access care within the dental tourism industry. One participant suggested that when he tried to coordinate with other clinic employees to develop a health promotion activity in local schools, no other clinic employees would work with him as they were sceptical about collaborating with a competitor and because they did not have the time to devote to this project. He explained that:

It is not common at all to find dentists who are interested in seeing local people, we are a small group of people who procure the local people, to treat them, and to charge less money for our services. Lowering our prices to serve them is difficult but I think it is possible and it can be done. I do it. (municipal tourism planner).

This participant expressed frustration with the culture of care in the dental tourism industry compared to care provision outside of the industry, explaining that: “We all are capable of providing preventive services, it is our duty as dentists to provide preventive services to the community. However, we unfortunately turn a blind eye to this situation here”. Overall, while some dentists and industry employees had developed initiatives to promote improved oral health amongst marginalized populations (i.e., one dentist uses his 3D printer to make crowns and molds for clinics in southern parts of Mexico where there are limited numbers of dental labs), multiple participants suggested these efforts were few and far between and were hard to pursue within the dental tourism industry environment focused on maximizing profits.

## Discussion

Our interview discussions with dentists working in the dental tourism industry in Los Algodones build on and nuance theories suggesting that there are potential negative health equity impacts of medical tourism industry development [10]. Our case study highlights how barriers to accessing dental care for populations living in Los Algodones and the surrounding region are entrenched through industry development as dental clinics focus on treating foreign patients to maximize their profits. Furthermore, our interviews suggest that dental services in the public sector are generally understood to be under resourced and insufficient to meet the oral health needs of local populations. Our findings indicate that neoliberal assumptions underpinning dental tourism industry development in Los Algodones help to justify this under resourcing of the public sector as the private provision of dental care is normalized and legitimized through a discourse of empowerment. We argue in the remainder of this section that this discourse of empowerment serves to mask and dismiss concerns raised by participants and highlighted by the experiences of particular residents related to standards of care and barriers to accessing care. We suggest that this discourse enables and even encourages ongoing industry development with limited regulatory oversight despite ethical and safety concerns for industry practices. Through an examination of how and why this discourse is taken up by industry stakeholders, we argue that this case study outlines contextual factors enabling industry development despite ethical concern.

Participants’ portrayals of the dental tourism industry in Los Algodones aligned closely with media coverage by suggesting that the context of care provision in this particular location enables international patients to access high quality dental care at more affordable prices than typically available in Canada and the US [23]. Many participants spoke positively about consumer-driven forms of regulation of the dental tourism industry in Los Algodones, such as patient review websites, and suggested that the use of these websites by prospective dental tourists provided these patients with information to choose more desirable care options. However, some participants’ stories and concerns about practices related to review websites contradicted these assertions: they expressed frustration at unfair reviews from patients who were unhappy with their care for reasons unrelated to the care itself. These examples align with findings in the literature that suggest that too much choice can be overwhelming and confusing to patients, specifically when this information is provided online [16]. We found that patients sometimes sought out unnecessary treatment in Los Algodones according to recommendations from other patients via word-of-mouth or as a result of confusion when navigating contradictory advice. While participants recognized and called out substandard or unfair practices in the industry, many also took up a discourse of empowerment to dismiss or legitimize any concerns related to patient safety and unethical practices.

Our findings align with research on patient review websites that indicate that within a context of profit-driven care provision, providers often feel pressured to adjust their practice to maintain their reputation online and continue to attract customers. While this adjustment might empower patients to find the right care provider for their needs, it also raises concerns about whether or not attention to customer satisfaction aligns with professional standards of care and ethical care provision [29]. Our research suggests that within a profit-driven industry, dentists often felt pressured to meet patient demands and at times, this pressure encouraged unethical and/or substandard care practices characterized by unlicensed providers, limited follow-up care, and unnecessary treatment. While the addition of care choices empowered patients in Los Algodones as consumers, this shift of power away from providers competing for these consumers’ dollars seemingly came at a cost to patient safety and responsible use of health resources. We suggest that this shift of power occurs as a result of competition for customers within the global medical tourism industry as industry development responds to the demand for more affordable care from patients in the global north.

Furthermore, our research indicates that pressure to meet the demands of dental tourists is produced in a context of limited economic opportunities. For example, many participants suggested that the industry provides work opportunities for dentists struggling to secure adequate employment in the public or private sector, a perspective that is reflected in the research on the dental industry in Mexico [30]. Research examining the perspectives of doctors working in the medical tourism industry in India found that many of these doctors felt as though they could access better professional development opportunities, wages, and labour conditions by treating international patients and, as a result, were willing to go along with certain industry protocols and norms to continue working in this context [6]. Similarly, our findings suggest that dentists felt they had limited acceptable employment options outside of the industry, and as a result, they had no choice but to take up certain practices that are highly encouraged throughout the industry, including lowering their prices, speeding up their care, and focusing on treatment which meets the needs of the dental tourist population. We found that many participants were also wary about introducing regulatory measures that might increase the costs of care provision while trying to attract customers within a competitive global industry. The empowerment generated by medical tourism is thus limited for practitioners in that they may become trapped by the demands of a highly competitive industry.

Finally, we found that many participants ignored or dismissed concerns about unjust or unsafe practices in the industry by distancing the work they perform in the medical tourism industry from health care provided within the formal health care system. This finding builds on research on “border doctors” working in ‘medical border towns’ which suggests that care providers might perceive themselves as working in a unique space along the Mexico-US border, outside of the national health care system. Within this context, providers might feel they do not have a responsibility to address local health needs as they are working in a tourism industry, not the health care system ([7], p., 1849). Many of our participants dismissed concerns regarding the exclusion of local populations from care provision in clinics catering to dental tourists despite oral health needs amongst local populations as populations struggle to access adequate dental care in contexts of poorly equipped public clinics and limited dental resources in rural areas [30]. Participants’ dismissal of unmet oral health needs in the local population corresponds with other research on medical tourism that suggests that many industry stakeholders perceive medical tourism activities to be a form of tourism diversification and/or entrepreneurial pursuit, distancing the work in the industry from the objectives and values regarding health equity underpinning the national health care system [18]. Thus, our research shows how industry stakeholders focused on the economic development potential of the industry might choose to focus on the empowerment experienced by some patients and providers, even though this empowerment occurs at a cost for global health equity and local access to care.

We suggest that this research nuances our understanding of how structural factors shape local medical tourism practices, including unethical or unsafe practices. As our findings show, patients are typically able to access care in the “escape valve” of Los Algodones’ dental tourism industry according to global relations of power ([7], p., 1847). As care providers focus on providing faster care at lower prices while protecting their reputations in online reviews, industry employees in Los Algodones avoided treating patients who cannot pay out of pocket for care, take time off work, or travel to this specific industry site [31], suggesting that this industry empowers the already empowered. Pressure on industry stakeholders to compete for customers and secure employment encouraged care provision that is more accessible and affordable to international dental tourists while further limiting access to this same care for marginalized populations. We argue that access to dental care barriers in Los Algodones were exacerbated through exclusionary practices at clinics catering their care to foreign patients and through the circulation of a discourse of empowerment assuming everyone who wants to access care can do so within Los Algodones’ dental marketplace. This discourse seemingly legitimized limited investment and attention to dental care for local residents while justifying ongoing industry development in ways that perpetuate and extend health inequities.

This study and the transferability of these findings are limited by several factors. First of all, our research site of Los Algodones is confined to a small border town, limiting the ability to draw inferences from this case study about the medical tourism industry in urban sites. The rurality of Los Algodones influenced the central importance of the dental tourism industry and reinforced the vulnerability of many local residents reliant on their employment within a competitive global industry. Furthermore, as a site located near the Mexico-US border, a border renowned for dividing the global north from the global south [32], this particular location of the industry site might limit the transferability of findings to our understanding of other sites and the medical tourism industry more broadly. For example, the location of Los Algodones near the Mexico-US border informed specific concerns raised in this research about patients pressing to have the work completed faster and at a lower cost, particularly given the history of American and Canadian snowbirds traveling across the border to shop for items at lower costs than available in their home countries [33].

Selection bias is also a possible limitation of this work [34]. KA primarily recruited participants by walking from clinic to clinic and knocking on doors. While we tried to invite participants representing various roles in the industry and from different backgrounds, doing so was limited by who was willing to speak with us. For example, we may have missed recruiting participants from clinics located in less obvious or accessible areas. Participants were also invited to participate through snowball sampling which may have limited the breadth of information or perspectives contributing to our analysis.

## Conclusion

In the context of Los Algodones’ dental tourism industry, the borderlands prove to be “quintessential spaces of exception wherein laws are regularly suspended” ([35], p. 211). As our research demonstrates, many dentists working in Los Algodones perceived themselves as “border doctors” ([7], p. 1849) working outside of the Mexican health care system. We argue that within this discourse of exceptionalism, industry stakeholders with a vested interest in the profitability of the industry work to portray this exceptionalism as opening up new possibilities for patient empowerment and economic development; however, we raise concerns about this discourse when it is used to legitimize unethical practices within the industry and to mask negative health equity implications of the industry. This discourse assumes patients can choose to purchase care within this “safety zone” as a form of empowerment and those who do not are failing to take responsibility for their care [7, 8]. Furthermore, within a context of a competitive global industry, industry stakeholders might feel they actually have no choice but to participate in practices meant to promote the industry and enhance patient choice, even when these practices do not align with their values as care providers and responsible patients. Overall, this analysis demonstrates that when taking up empowerment discourse, many industry stakeholders can ignore who is (not) empowered by the industry and how this type of empowerment might exacerbate health inequities, enabling ongoing industry development with limited regulatory oversight. Our case study of dental tourism in Los Algodones, Mexico suggests that industry practices and the language of empowerment used to promote the industry instead serve to trap patients and providers into practices aimed at profit maximization while widening global health inequities.

## Additional file

Additional file 1 Interview Guide: Los Algodones. (PDF 332 kb)
