# Supplementary material for: A critical examination of empowerment discourse in medical tourism: the case of the dental tourism industry in Los Algodones, Mexico
Source: Global Health. 2018 Jul 20;14:70. doi: 10.1186/s12992-018-0392-3 (PMC6054732; doi:10.1186/s12992-018-0392-3)
Supplement: Supplementary file 1 — Interview Guide: Los Algodones. (PDF 332 kb) [file 12992_2018_392_MOESM1_ESM.pdf]

Interview Guide: Los Algodones

1. How old are you? How long have you been living in Los Algodones?
    a. Where did you grow up?
    b. Why did you decide to become a dentist?
    c. Why did you decide to come practice in Los Algodones?
    d. Do you practice dentistry outside of Los Algodones as well?
    e. In your practice, what percentage of your patients are tourists and what percentage are residents that live in this area? Is that typical for most dentists working here?
    f. Ideally, where would you be working as a dentist if you could practice anywhere? Why?

2. How would you describe the oral health of people living in this region?
    a. How do you see dental care as improving oral health amongst people living in this region?
    b. How do you think people living in this area perceive dentistry and oral health? What are peoples' attitudes towards dental care?
    c. What changes do you think should be made to the provision of dental care in this region? Why?

3. Where did you do your dental training? Why did you decide to do your training there?
    a. Tell me more about your training? How long did it take? What were the costs like? How did your schooling talk about dentistry as a career? ie: options for where to work? Your roles and responsibilities to your community?
    b. How did your training prepare you specifically to treat dental tourists?
    c. Was there anything you wished you had learned in dental school? Why?

4. Can you tell me more about your work as a dentist here in Los Algodones? What is unique about working as a dentist in Los Algodones?
    a. What do you like about it? What don't you like about it?
    b. How do you deal with the competition in this town? How do you recruit patients? What are your strategies?
    c. How do you determine a treatment plan for the patient? Do most patients come with set treatment plans or do you provide diagnostic work as well?
    d. How much follow-up care do you provide? Do you ever send home records to communicate with patients' dentists back home?
    e. How do you determine what prices to charge for your care? Is there a fee guide? What costs do you have to consider? Are these prices different from prices elsewhere in Mexico?
    f. Why are prices so much different in Los Algodones than dental care provided in Canada?

5. How do you describe the cost differences to patients? How do you convince dental patients that lower costs of care do not indicate lower quality of care?
    a. What concerns do dental tourists typically have about accessing dental care in Los Algodones? What informs these concerns?

b. How do you try and provide confidence in your dental treatment to a dental tourist? How does this inform your marketing strategy? Is this different than how you might try and market your services to a non-tourist?
c. What information do you wish dental tourists knew about Los Algodones before coming here?

6. How is the quality of dental care in Los Algodones regulated?
    a. Are there any measures or assessments that determine if a dentist in Los Algodones is providing sub-standard care? What would happen to that dentist?
    b. Are there any professional organization or associations that regulate the quality of care provided by dentists in Los Algodones? In Baja California?
    c. How does regulation of health care differ between Los Algodones and elsewhere in Baja California?
    d. Are there any ethical codes of conduct or social responsibilities of dentists that you must follow? What does this entail? Do you have a social responsibility to improve the oral health of the community within which you are practicing?

7. In the future, do you think more dentists will establish businesses here? Why or why not?
    a. Should the industry expand and develop? Why or why not?
    b. How will the government, at the municipal, state, and national level, play a role in the potential development of the industry? What other stakeholders do you see as playing a major role in shaping how the industry develops?
    c. Will industry development or growth be positive for everyone living in this region? Who might not benefit from increasing industry growth? Why or why not?
